# Identification of a novel gross deletion of *TCOF1* in a Chinese prenatal case with Treacher Collins syndrome

**DOI:** 10.1002/mgg3.1313

**Published:** 2020-06-15

**Authors:** Jing Liu, Pengsiyuan Lin, Jialun Pang, Zhengjun Jia, Ying Peng, Hui Xi, Lingqian Wu, Zhuo Li, Hua Wang

**Affiliations:** ^1^ Department of Medical Genetics Hunan Provincial Maternal and Child Health Care Hospital Changsha Hunan China; ^2^ National Health Commission Key Laboratory of Birth Defects Research, Prevention and Treatment Changsha Hunan China; ^3^ Center for Medical Genetics & Hunan Key Laboratory of Medical Genetics School of Life Sciences Central South University Changsha Hunan China

**Keywords:** craniofacial malformation, prenatal diagnosis, targeted exome sequencing, *TCOF1*, Treacher Collins syndrome

## Abstract

**Background:**

Treacher Collins syndrome (TCS) is the most common mandibulofacial dysostosis with an autosomal dominant or rarely recessive manner of inheritance. It is still challenging to make a definite diagnosis for affected fetuses with TCS only depending on the ultrasound screening. Genetic tests can contribute to the accurate diagnosis for those prenatal cases.

**Methods:**

Targeted exome sequencing was performed in a fetus of a Chinese family, who presenting an abnormal facial appearance by prenatal 2D and 3D ultrasound screening, including micrognathia, nasal bridge pit, and abnormal auricle. The result was validated with multiplex ligation‐dependent probe amplification (MLPA) and real‐time quantitative PCR (qPCR).

**Results:**

A novel 2–6 exons deletion of *TCOF1* gene was identified and confirmed by the MLPA and qPCR in the fetus, which was inherited from the affected father with similar facial anomalies.

**Conclusion:**

The heterozygous deletion of 2–6 exons in *TCOF1* results in the TCS of this Chinese family. Our findings not only enlarge the spectrum of mutations in *TCOF1* gene, but also highlight the values of combination of ultrasound and genetics tests in diagnosis of craniofacial malformation‐related diseases during perinatal period.

## Graphical abstract

A novel gross deletion of 2–6 exons in TCOF1 was identified in both of the fetus and his affected father by targeted sequencing, and confirmed via multiplex ligation‐dependent probe amplification and real‐time quantitative PCR, providing a precise diagnosis of Treacher Collins syndrome and important information for genetic counseling on the family.

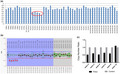

## 1 INTRODUCTION

Treacher Collins syndrome (TCS, OMIM 154500) is a rare craniofacial malformation that occurs with an estimated prevalence of 1/50000 live births. The minimal diagnostic criteria were defined as hypoplasia of the zygomatic arches and downslanting palpebral fissures (Teber et al., 2004). Other frequent findings include mandibular hypoplasia, colobomata of the lower eyelids, and ear malformations often associated with bilateral conductive hearing loss (Trainor & Andrews, 2013; Wong, Pfaff, Chang, Travieso, & Steinbacher, 2013). Most cases are inherited as an autosomal dominant trait, although an autosomal recessive form of this syndrome has been reported (Dauwerse et al., 2011; Schaefer et al., 2014).

Treacher Collins syndrome has been proven to be genetically heterogeneous, and three genes (*TCOF1*, *POLR1D,* and *POLR1C*) have been identified in the etiology of TCS. Among the causative genes, *TCOF1* (OMIM 606847) mutations are most common, which account for approximate 70%–93% of studied individuals with autosomal dominant cases (Bowman et al., 2012; Splendore et al., 2000). To date, more than 300 distinct mutations have been identified within the *TCOF1* gene and most of the mutations are family specific collected in the Human Gene Mutation Database^®^ (HGMD). However, pathogenic mutations in about 11% of TCS cases remain unknown (Chen et al., 2018).

A remarkable feature of TCS is the large amount of inter‐ and intra‐family variation in phenotype severity, and no genotype–phenotype correlation has been found based on the evaluation of the clinical variability in TCS (Dixon, Ellis, Bottani, Temple, & Dixon, 2004). It has been reported that TCS expressivity varies from non‐affected and mildly affected to severely affected individuals or perinatal deaths (Schlump et al., 2012). These factors contribute to the difficulties of definitive clinical diagnosis and genetic counseling for TCS patients, especially for those prenatal fetuses. Currently, prenatal TCS may be detected by means of conventional two‐dimensional (2D) ultrasound or by the more refined three‐dimensional (3D) scanning, however, it is rarely effective for mild to moderate TCS, particularly in sporadic cases (Hsu, Hsu, Chang, & Chang, 2002; Ruangvutilert, Sutantawibul, Sunsaneevithayakul, & Limwongse, 2003; Tanaka et al., 2002). Thus, identification of causative genes and mutations for the affected fetus is of great importance to precisely prevent TCS.

Here, we describe a 24‐week fetus with mandibulofacial dysostosis via ultrasound testing, who was clinically suspected to be TCS after physical examination for his father with similar abnormal facial features. A novel gross deletion of 2–6 exons in *TCOF1* was identified in both of the fetus and his father by targeted sequencing, and confirmed via multiplex ligation‐dependent probe amplification (MLPA) and real‐time quantitative PCR (qPCR), providing important information for genetic counseling on this family. To our knowledge, this is the first report on the genetic diagnosis for a fetus with TCS in Chinese population.

## 2 CLINICAL REPORT

**FIGURE 1:**
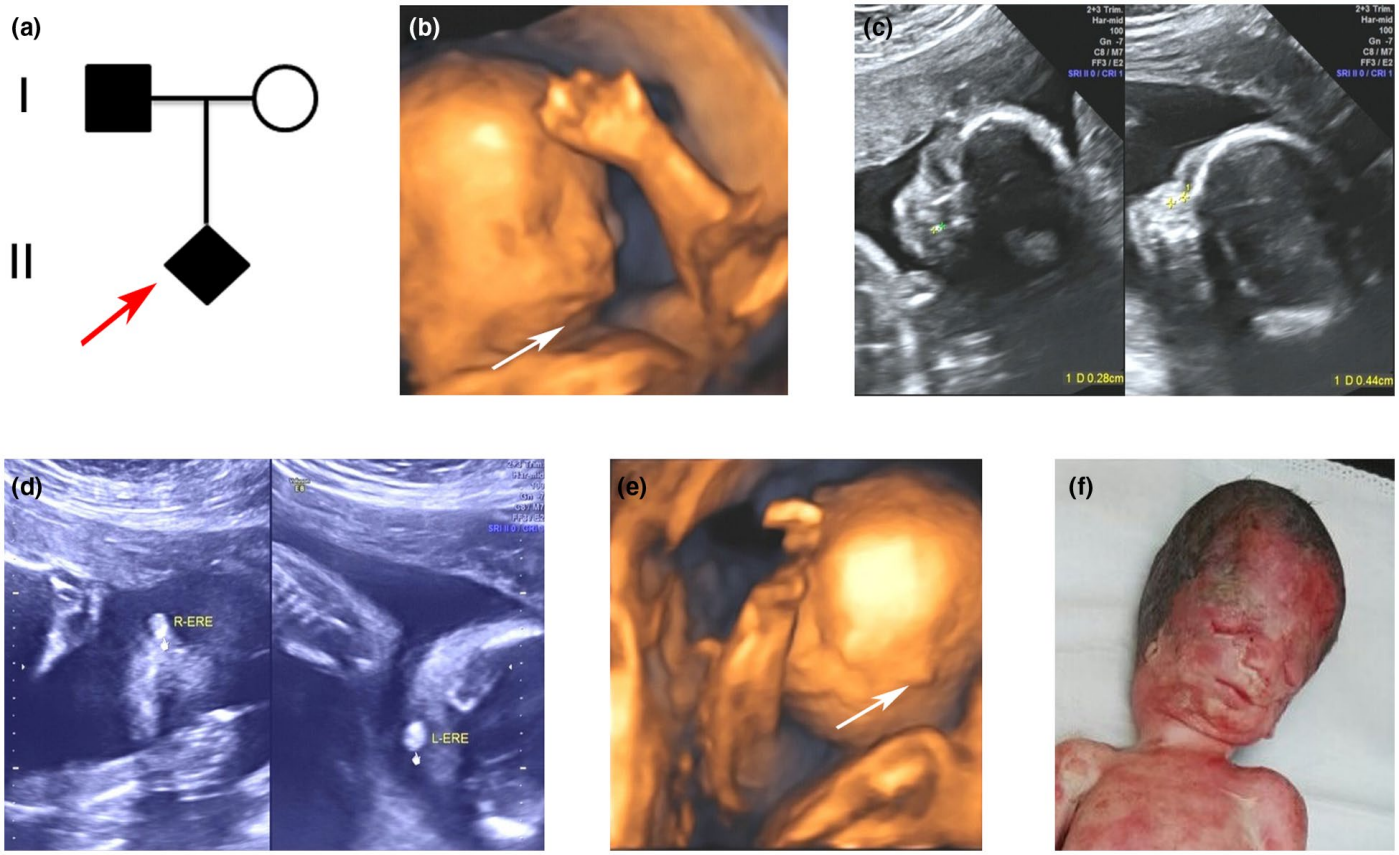
Family pedigree and male fetus at 24 weeks of gestation with craniofacial features by 2D and 3D ultrasound inspection. (a) Family pedigree. Red arrow indicates the proband. (b) Micrognathia was displayed in 3D ultrasound image. (c) Asymmetric nasal bone with 0.28 cm in left side and 0.44 cm in right side was detected. (d and e) Extremely small ears in both sides were screened by 2D and 3D ultrasound. (f) The craniofacial malformation of the aborted fetus at 25 weeks with slanting palpebral fissures, coloboma of the eyelid, hypoplastic zygomatic arches, and abnormal ears

A gravid 1, para 1 woman came to our clinical genetics center for genetic counseling at 24 weeks of gestation. This is her first pregnancy (Figure 1a). A first round prenatal two‐dimensional and three‐dimensional ultrasound revealed that the male fetus (II:1) showed multiple defects, including micrognathia (Figure 1b), asymmetric nasal bone with 0.28 cm in left side and 0.44 cm in right side (Figure 1c), as well as abnormal auricle in both sides with only 0.76 cm and 0.67 cm high echo sound (Figure 1d,e). Other indicators of the fetus seemed normal, with biparietal diameter of 6.1 cm (50–90th centile), head circumference of 22.7 cm (50–90th centile), abdominal circumference of 19.7 cm (50–90th centile), femora length of 4.3 cm (50–90th centile),and humerus length of 4.0 cm (50–90th centile).

The mother (I:2) was 35 years old and her husband (I:1) was 39 years old. Both of them were non‐consanguineous Chinese. When her husband appeared in our center, we found that he also had an abnormal facial appearance, with slanting palpebral fissures, coloboma of the eyelid, hypoplastic zygomatic arches, but without atresia of the external ear canal and cleft palate. A hearing loss of left side was also mentioned. They denied a family history with same phenotypes.

Based on the clinical features of the fetus and his father, a clinical diagnosis of TCS was highly suspected. Therefore, the family was referred for molecular diagnosis. Fetal cord blood and peripheral blood from the parents were obtained. After sufficient genetic counseling and totally informed the prognosis of TCS, the parents still decided to terminate the pregnancy at 25 weeks. The craniofacial features of the aborted fetus were consistent with the ultrasonic findings and additional down‐slanting palpebral fissures were discerned Figure 1f), but further autopsy was refused.

## 3 TARGETED EXOME SEQUENCING FOR SKELETAL SYSTEM DISEASE GENES

The patients provided an informed consent to publish all clinical information. The research was approved by the Ethics Committee of Hunan Provincial Maternal and Child Health Care Hospital. DNA was extracted from blood samples of the family. A minimum of 3 μg DNA was used to create the indexed Illumina DNA libraries, according to the manufacturer's protocol. All the exons of the 248 genes known to be involved in skeletal system diseases were enriched using the GenCap custom enrichment kit (MyGenostics Inc.). The enriched exome libraries were sequenced on an Illumina HiSeq 2000 sequencer (Illumina), using the paired‐end method, for the 100‐bp reads.

Data analysis: The Trim Galore and Solexa cutadapt programs were used to filter out the low‐quality reads and adapter sequences. Only the reads with a sequencing quality >20, a depth >90, and a read length >80 bp were retained. The clean reads were aligned to each human reference genome (hg19) using the SOAPaligner program (BGI). The duplicated reads were removed using the Sequence Alignment/Map tools (SAMtools) V3 and only the uniquely mapping reads were used for detection of variation. The single nucleotide variants (SNVs) were detected and genotyped with the SOAPsnp program (BGI). Subsequently, a Burrows–Wheeler Aligner (BWA) was used to realign the reads to the reference genome. The insertions or deletions (InDels) were identified using the GATK software package. The identified SNPs and InDels were annotated using multiple databases, including the Consensus CDS database, the human genome builder NCBI 37, the dbSNP database V147, and the In‐house sequencing data of 300 local normal Asians.

## 4 MLPA AND QUANTITATIVE PCR DETECTION

The SALSA MLPA P310 *TCOF1* probe mix kit (MRC‐Holland) was performed to determine the DNA copy number of a single DNA sequence in *TCOF1* regions and 10 different autosomal chromosomal locations. MLPA analysis was performed following the manufacturer's instructions. The products were examined by ABI 3500dx genetic analyzer (Thermo Fisher). Quantitative data were analyzed using the software of Coffalyser V8.0. Validation of the result was carried out by qPCR (Primers are listed in Table S1).

## 5 RESULTS

**FIGURE 2:**
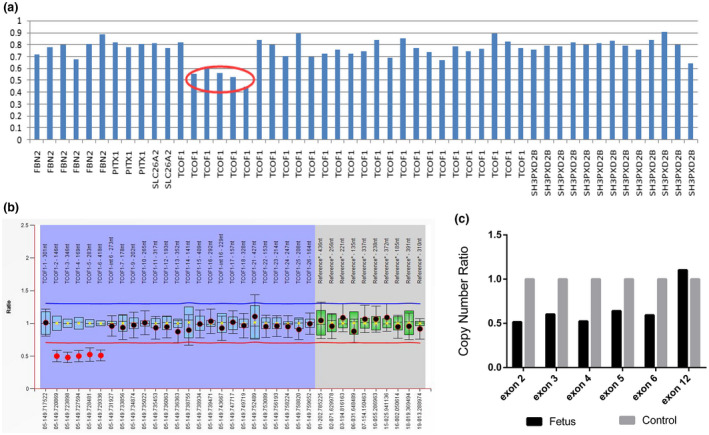
Identification of a gross deletion of *TCOF1* in the fetus by genetic testing. (a) Targeted exome sequencing revealed a heterozygous deletion of 2–6 exons of *TCOF1* as indicated by the red circle. (b) MLPA also showed a 50% relatively decreased bar height of 2–6 exons in *TCOF1* gene, indicating a heterozygous deletion. (C) qPCR confirmed a half‐dose for 2–6 exons of *TCOF1* in the affected fetus compared with the normal individual. Exon 12 was set up as a normal control. MLPA, ligation‐dependent probe amplification; qPCR, real‐time quantitative PCR

As the clinical manifestations of the father highly match the typical features of TCS, fetal, and paternal DNA samples were examined by targeted exome sequencing (cover 248 genes known to be involved in skeletal system diseases), which would detect not only the SNVs, but also large indels among the targeted genes according to the optimized bioinformatics analysis. After targeted exon sequencing, three candidate mutations in different genes were screened out, including a novel heterozygous deletion of 2–6 exons in *TCOF1* (NM_001008657.3), two heterozygous missense mutations of c.2654G>A/p.R885Q and c.2482C>G/p.Q828E in *EVC2* (NM_147127.4) and *FLNB* (NM_001457.2), respectively. According to further genotype–phenotype analysis, we found that heterozygous mutations of *EVC2* gene are responsible for Weyers acrofacial dysostosis (OMIM 193530), which were mainly characterized by mild short stature, postaxial polydactyly, and so on. The *FLNB*‐related disorders include a spectrum of phenotypes, including atelosteogenesis types I (OMIM 108720), type III (OMIM 108721, Larsen syndrome (OMIM 150250), and Boomerang dysplasia (OMIM 112310), but these disorders are mainly characterized by either severe short limbs, or dislocations of the hip, knee, and elbow, or underossification of the limb bones and vertebrae, etc. However, the proband and his father in our case did not show any malformation in their trunk and limbs, except the craniofacial abnormalities, thus both *EVC2* and *FLNB* are unlikely to be the genetic cause for the family. Actually, the clinical features of the family highly match the TCS caused by *TCOF1* mutations, and based on the mode of inheritance, mutation frequency, and pathogenicity prediction through multiple softwares, the novel heterozygous deletion of 2–6 exons in *TCOF1* was thereby classified as a pathogenic variant according to the ACMG (the American College of Medical Genetics and Genomics) guideline (Figure 2a) (Richards et al., 2015). The deletion was then confirmed by MLPA and qPCR testing. Both results revealed that the copy number of 2–6 exons was only half of other exons in *TCOF1,* suggesting a heterozygous deletion in 2–6 exons (Figure 2b,c). This gross deletion has not been reported yet, nor recorded in related databases. Cosegregation analysis demonstrated that the fetus inherited the same deletion from his affected father, while the mother showed normal genotype in *TCOF1*. Therefore, the affected fetus and his father were molecularly diagnosed to be the TCS, due to the pathogenic mutation of 2–6 exons in *TCOF1*.

## 6 DISCUSSION

Treacher Collins syndrome exhibits great phenotypical variability ranging from perinatal death to clinically undiagnosed cases. When structural anomalies are present in prenatal cases, usually it is a task for the sonographer to first clinically diagnose a genetic syndrome due to incomprehensive information from the imaging detection, especially for those sporadic cases. Although TCS is characterized with craniofacial malformation, several syndromes involved with facial dysmorphism have been described, among which the main differential diagnoses of TCS are with the Goldenhar and Nager syndromes. Main symptoms of Goldenhar syndrome include hemifacial microsomia, incomplete development of the ear, cleft lip and/or palate, ocular, and vertebral abnormalities (Barisic et al., 2014). Nager syndrome is characterized by malar hypoplasia, ear abnormalities, micrognathia, downslanting palpebral fissures, radial hypoplasia of the limbs, and absence of thumbs, and/or other fingers (Lansinger & Rayan, 2015).

In our case, the male fetus presented with facial malformations, including micrognathia, abnormal nose, ear anomalies, and suspected downslanting palpebral fissures by the first round 2D and 3D ultrasound. Although limbs and fingers of the fetus were normal, it was still hard to differentially define a specific syndrome only depending on the ultrasound results. Until the father came to our genetic outpatient at the second visiting, displaying visible slanting palpebral fissures, coloboma of the eyelid, hypoplastic zygomatic arches, the father, and fetus were highly suspected to be suffered from TCS, as there is no additional hemifacial microsomia or limbs abnormalities, which usually presenting in Goldenhar syndrome or Nager syndrome.

Among known causative genes of autosomal dominant TCS, *TCOF1* is responsible for approximately 70%–93% of studied individuals (Dauwerse et al., 2011). *POLR1D* and *POLR1C* mutations account for 11%–23% of patients without *TCOF1* mutations (Fan et al., 2019). *TCOF1* encodes for a protein called treacle, which is part of a complex implicated in ribosomal RNA biogenesis and is involved in the proliferation and differentiation of neural crest cells in the first and second branchial arches during early embryogenesis (Dixon et al., 2006). To date, more than 300 different mutations of *TCOF1* gene have been reported in patients with TCS according to HGMD data. The mutations include missense, nonsense, small deletions, and duplications. In particular, the most common classes of *TCOF1* alleles are small deletions (60%) and duplications (25%) resulting in frameshifts (Conte et al., 2011). Though it has been suggested that five exons (10, 15, 16, 23, and 24), are defined as a hot spot region of the *TCOF1* gene mutations (Splendore, Fanganiello, Masotti, Morganti, & Passos‐Bueno, 2005), a distribution of pathogenic variations along all the gene was reported by different authors.

Large deletions encompassing the *TCOF1* gene or gross deletions in *TCOF1* were rare in literature (Table 1) (Beygo et al., 2012; Bowman et al., 2012; Vincent et al., 2014, 2016). Beygo et al. (2012) reported the first single exon deletion within *TCOF1*. Another study conducted by Bowman et al described five pathogenic rearrangements in *TCOF1*, which suggested that *TCOF1* rearrangements are responsible for a significant proportion (5.2%) of TCS‐associated mutations, and they therefore recommended that dosage analysis by MLPA or a comparable method should be undertaken as part of a *TCOF1* gene screening service (Bowman et al., 2012). Vincent et al reported on two patients presenting with mandibulofacial dysostosis characteristic of TCS, associated with unexpected intellectual disability, due to a large deletion encompassing several genes including the *TCOF1* gene (Vincent et al., 2014, 2016).

**TABLE 1:** Previously reported gross deletion mutations in patients with TCS

Patients	Deletion Region	Clinical manifestations	References
Patient 1	Exon 1‐15 (*TCOF1*)	mandibular hypoplasia, cleft palate, down‐slanting palpebral fissures, wide set eyes, and abnormal palmar creases	Bowman et al. (2012)
Patient 2	Exon 1 (*TCOF1*)	clinical diagnosis of TCS	
Patient 3	3′‐UTR (*TCOF1*)	Mild TCS	
Patient 4	Exon 23‐25, 3′‐UTR (*TCOF1*)	clinical diagnosis of TCS	
Patient 5	Exon 1–6 (*TCOF1*)	clinical diagnosis of TCS	
Patient 6	Exon 3 (*TCOF1*)	down‐slanting palpebral fissures, a right‐sided lower eyelid coloboma with absence of eyelashes medial to the defect, hypoplasia of the zygomatic complex as well as conductive deafness and median cleft palate	Beygo et al. (2012)
Patient 7	*1Mb incl TCOF1, CAMK2A+*14 others	Atypical TCS (Severe): down‐slanted palpebral fissures with mild colobomatous cleft of the lower lid, small mandible with class III malocclusion, and low‐set and dysplastic ears	Vincent et al. (2014)
Patient 8	*262kb incl TCOF1, CAMK2A+*14 others	Atypical TCS (Severe): down‐slanted palpebral fissures, malar hypoplasia, micrognathia and microtia, and mild ID.	
Patient 9	Exon 11 (*TCOF1*)	Atypical TCS (Mild)	Vincent et al. (2016)
Patient 10	5′‐UTR, exon 1–2 (*TCOF1*)	Typical TCS (Severe)	
Patient 11	Exon 24 (*TCOF1*)	Typical TCS (Mild)	
Patient 12	Exon 25 (*TCOF1*)	Typical TCS (Severe)	
Patient 13	Exon 9–13 (*TCOF1*)	Typical TCS: Microtia with atretic external auditory canals on the right side. Mandibular retrognathia, downward slanting palpebral fissures, and coloboma of the lower eyelids were also evident at birth	Li et al. (2019)
Our case	Exon 2–6 (*TCOF1*)	Typical TCS: slanting palpebral fissures with coloboma of the eyelid, hypoplastic zygomatic arches	Present report

Abbreviation: TCS, Treacher Collins syndrome.

According to previous studies, most mutations reported in Chinese TCS patients were in *TCOF1* gene. Majority of these pathogenic variations were SNVs and small Indels ranging in size from 1 to 38 nucleotides, only one gross deletion (exon 9–13) was reported before (Table S2) (Chen et al., 2018; Fan et al., 2019; Li et al., 2012, 2019; Wang et al., 2014; Yan et al., 2018; Zhang, Fan, Zhang, Xue, & Chen, 2013).

In this study, we performed targeted exome sequencing on the family with a requirement for prenatal diagnosis, due to the high suspicion of TCS for the fetus and his father. A novel gross deletion of 2–6 exons in *TCOF1* was identified and confirmed the diagnosis of TCS for the family. This gross deletion was never reported in literatures and might lead to loss of protein function consistent with a mechanism of haploinsufficiency. Regarding to the genotype–phenotype correlation, a previous report with a large cohort has proved that although *TCOF1* has a LisH domain from exon 1 to 2 and a Treacle domain from exons 2 to 24, the localization of the mutations in the LisH or Treacle domains had no different effects on clinical features or severity of TCS (Vincent et al., 2016). In our case, we also found no special phenotypes compared with other patients with different mutations.

Molecular diagnosis plays a significant role for patients with TCS, in both prenatal and postnatal stages, and has an undeniable impact on the development of genetic counseling. In this case, we combined 3D ultrasound and targeted exome sequencing together to identify a TCS fetus successfully. A novel gross deletion of 2–6 exons in *TCOF1* was ascertained in this Chinese family, which enlarged the spectrum of mutations in *TCOF1* gene. Our study also indicates that when facing with a similar case with craniofacial malformation in prenatal stage, molecular genetics testing would help a lot to make definite diagnosis to guide the following genetic counseling.

## CONFLICT OF INTEREST

The authors declare that they have no competing interests.

## AUTHORS’ CONTRIBUTIONS

Jing Liu, Zhuo Li, and Hua Wang had major roles in the design of the study. Jing Liu and Zhuo Li drafted the manuscript. Jing Liu, Pengsiyuan Lin, Jialun Pang performed the molecular genetic experiments and analyzed the data. Zhengjun Jia, Ying Peng, Hui Xi, and Lingqian Wu analyzed the clinical data. Zhuo Li and Hua Wang are corresponding authors of this manuscript. All authors read and approved the final manuscript.

## Supporting information

Table S1‐S2

## Data Availability

The data that support the findings of this study are available from the corresponding author upon reasonable request.
