# Supplementary material for: Identification of a novel gross deletion of TCOF1 in a Chinese prenatal case with Treacher Collins syndrome
Source: Mol Genet Genomic Med. 2020 Jun 15;8(8):e1313. doi: 10.1002/mgg3.1313 (PMC7434750; doi:10.1002/mgg3.1313)
Supplement: Supplementary file 1 — Table S1‐S2 [file MGG3-8-e1313-s001.docx]

Supplemental Table 1. Primers for qPCR

| **Primers** | **Sequences (5'to3')** |
| --- | --- |
| TCOF1-2F | CCTCTTCTGAACCACCTGTC |
| TCOF1-2R | CGTCCCTACTCCACCCTAT |
| TCOF1-3F | GATGCGGGAAGGTCTGT |
| TCOF1-3R | ACTGGCTTTGGCGGTTT |
| TCOF1-4F | GGCAGCCAATACCAATAGAA |
| TCOF1-4R | CCTTAAATCCCATAGGCAATA |
| TCOF1-5F | GTGGCCTGTGCTCTTAGTTCA |
| TCOF1-5R | TGCCCTCCTCCTCAGTTTC |
| TCOF1-6F | CTCCGTGTCCGATCCTCA |
| TCOF1-6R | GTCACCCTACCACAGTTGCT |
| TCOF1-8F | ATCACCAGAGAGTTTTCACAAGCA |
| TCOF1-8R | CCTTGGTCTGGGAGGCTACA |
| TCOF1-12F | GCCAAACCCACCTCCAGT |
| TCOF1-12R | CATGTAGCAGCCTCCTTCC |

Supplemental Table 2. Previously reported mutations of *TCOF1* in Chinese patients with TCS

| Authors | HGVS | Protein | Exon |
| --- | --- | --- | --- |
| Li et al. (2012) | c.1639_1640 delAG | p.S547Qfs*2 | Exon 10 |
| Zhang et al. (2013) | c. 4369_4373delAAGAA  c.4420C>T | p.K1457Gfs*12  p.Q1474* | Exon 24  Exon 24 |
| Wang et al. (2014) | c.1303_1304insC  c.2103_2106delTGAG  c.1658C>G | P.Q45Pfs*23  P.Q45Pfs*23  p.S553* | Exon 9  Exon 11  Exon 10 |
| Chen et al. (2018) | c.136C>G | p.L46V | Exon 2 |
|  | c.159G>A | p.W53* | Exon 2 |
|  | c.430_431insA | p.T144Nfs*31 | Exon 5 |
|  | c.451delC | p.L151Ffs*68 | Exon 5 |
|  | c.810_811insA | p.E271Rfs*3 | Exon 6A |
|  | c.1155_1160delAGCTGC  insGGGACTT | p.A386Gfs*35 | Exon 8 |
|  | c.1307_1344del | p.V436Efs*9 | Exon 9 |
|  | c.1719_1720insG | p.N574Efs*29 | Exon 11 |
|  | c.3386delA | p.K1129Sfs*79 | Exon 20 |
|  | c.3496delG | P.A1166Pfs*42 | Exon 20 |
|  | c.4129_4130delGT | p.V1377Ffs*21 | Exon 23 |
|  | c.3823delC  c. 4369_4373delAAGAA | p.R1275Gfs*32  p.K1457Gfs*12 | Exon 23  Exon 24 |
|  | c.4231C>T | p.Q1411* | Exon 23 |
| Yan et al. (2018) | c.165-1G>A | p.(?) | Exon 3 |
| Fan et al. (2019) | c.3047-2A > G | p.(?) | Intron 17 |
|  | c.2478 + 5G > A | p.(?) | Intron 14 |
|  | c.489delC | p.S164Qfs*55 | Exon5 |
|  | c.648delC | p.S217Qfs*2 | Exon6A |
| Li et al. (2019) | c.4131_4135delAAAAG | p.K1380Efs*12 | Exon24 |
|  | c.2394_2395delAG | p.D799Qfs*2 | Exon15 |
|  | c.381_382 delAG | p.A127fs46* | Exon5 |
|  | Exon9-13 deletion | p.(?) | Exon9-13 |
